# Use of various obesity measurement and classification methods in occupational safety and health research: a systematic review of the literature

**DOI:** 10.1186/s40608-018-0205-5

**Published:** 2018-11-01

**Authors:** Mahboobeh Ghesmaty Sangachin, Lora A. Cavuoto, Youfa Wang

**Affiliations:** 1 Department of Industrial and Systems Engineering, University at Buffalo, 324 Bell Hall, Buffalo, NY 14260 USA; 2 Department of Nutrition and Health Sciences, College of Health, Ball State University, Muncie, IN USA

**Keywords:** Obesity, Overweight, Body mass index, Occupational safety and health, Ergonomics

## Abstract

**Background:**

This study systematically examined obesity research in occupational safety and health regarding the use of various obesity measurement and classification methods.

**Methods:**

A systematic search of the PubMed database on English language publications from 2000 to 2015 using related keywords and search of citations resulted in selection of 126 studies. They were categorized into two groups based on their main research question: 1) general physical or mental work-related functioning; and 2) task or body part specific functioning.

**Results:**

Regardless of the study group, body mass index (BMI) was the most frequently used measure. Over 63% of the studies relied solely on BMI to define obesity. In only 22% of the studies, body fat was directly measured by methods such as dual energy x-ray absorptiometry. Abdominal obesity was defined using waist circumference in recent years, and waist-hip ratio in earlier years. Inconsistent cut-offs have also been used across studies investigating similar topics.

**Conclusions:**

Few authors acknowledged the limitations of using indirect obesity measures. This is in part due to the limited understanding of some occupational safety and health researchers regarding the complex issues surrounding obesity classification and also the mixed recommendations over the past 2–3 decades and across populations. Efforts need to be made to promote appropriate obesity measurement and reporting in this field.

## Background

Obesity affects over 600 million adults worldwide and the number continues to grow [1]. Along with the rise in its worldwide prevalence [2, 3], the evidence for its adverse effects on individuals’ health has been accumulating. Obesity has been identified as a risk factor for cardiovascular disease [4], pulmonary embolism [5], large joint osteoarthritis (OA) [6], and certain types of cancer [7]. It has also been associated with a decrease in general physical function [8], as well as cognitive abilities [9]. The diversity in the adverse outcomes attributed to obesity, the complexity in the mechanisms leading to them, and the multi-factorial nature of this disease require the joint effort of different scientific disciplines to better understand the scope of the problem and to limit its detrimental effects.

With the prevalence of obesity among the workforce being equal to that of the general population [10], the occupational safety and health discipline has shown interest and effectively contributed to obesity research. The effects of obesity on work performance, physical capacity, and physical and cognitive function have been the research focus of many ergonomists, work analysts, and occupational health experts. As such, employees who are obese have been found to have higher rates of sick leave [11] and workplace injuries [12], along with increased employer-paid healthcare costs [13]. As these efforts expand to evaluate the relation between obesity and work [14], it is essential to explore how obesity status is measured in this field (e.g., body mass index (BMI) and body fat percentage (%BF)) as well as the basis for classifying individuals into distinct risk groups (e.g., types I and II obesity). In general, the issue of obesity measurement is two-fold: 1) selection of the appropriate measurement and 2) properly carrying out the measurement process to minimize measurement error.

### Measurement selection

The World Health Organization (WHO) defines obesity as abnormal or excessive fat accumulation that may impair health [15], and this definition should serve as the basis for measurement selection. While underwater weighing and dual energy x-ray absorptiometry (DEXA) directly measure body fat, many indirect measures of adiposity have been used to measure obesity status. Anthropometric measures such as the weight-for-height index, BMI, waist circumference (WC), waist–hip ratio (WHR), and body fat percentage estimated by skinfold thickness (ST) are widely accepted indirect measures. Since the 1990s, BMI has been widely used to classify overweight and obesity, both in adults and children [16]. BMI has been suggested as an ideal measure of adiposity since it is easy to measure and is closely associated with obesity related health risks [17].

However, indirect measures such as BMI, fail to distinguish between fat, muscle or bone mass and are prone to misclassification, particularly among muscular subjects [18]. Mullie et al. [19] compared %BF, measured by bipolar bioelectrical impedance analysis (BIA), and BMI, for a cohort of 448 male military candidates and found a statistically significant difference between classifications of normal weight versus overweight. Almost 40% of the subjects classified as overweight with BMI > 25 kg/m^2^ had a %BF corresponding to normal weight. Similarly, Deurenberg et al. [20] observed a higher rate of misclassification with BMI compared to DEXA in 416 European individuals. This study showcased how individual results based on a single classification method should be interpreted with caution.

Reliance only on BMI can also lead to misclassification of those with excess body fat, but BMIs corresponding to normal weight. These “metabolically obese but normal weight” [21] individuals share many health risks with those categorized as obese both based on BMI and %BF [22]. The elevated visceral fat observed in this category is accompanied by increased levels of both liver and muscle fat [23]. In a workplace study, comparing new industry hires from 1990 to 1992 and from 2000 to 2002, there was no significant difference in BMI but a significant difference in %BF, measured by ST [24]. There were also significant differences in physical fitness as measured by timed sit-ups and squats, suggesting that employers would miss information regarding their employees’ fitness with reliance on BMI only. BMI is also not independently representative of body fatness. Significant dependencies on age and sex were observed in the relation between %BF and BMI in a study of 706 adult men and women [25]. BMI also overlooks the distribution of fat, which is an important factor in disease risk. For instance, android fat distribution (also referred to as abdominal, central, visceral, or upper body fat distribution) causes increased risk of diseases such as cardiovascular disease and type 2 diabetes [26], while gynoid fat distribution (i.e. larger hip and thigh circumferences) does not seem to have similar effects [27]. Indices such as WC and WHR are useful in characterizing the obesity morphology, particularly for studies where a difference in anthropometry among subjects is relevant to consider.

Misclassification and measurement error may be exacerbated in small sample sizes, which are common in exploratory laboratory-based occupational safety and health studies. Piers et al. [28] showed that despite the significant correlation between BMI and %BF (measured by underwater weighing method) of the 117 healthy samples, BMI only explained, on average, 50% of the variance in %BF. The reported poor sensitivity (47.7%) and positive predictive value (67.7%) of BMI makes it an unreliable measure of obesity in individuals. These findings not only suggest the inadequacy of BMI in classification of obesity status, particularly for individuals near the cutoff values, but also point out the importance of a rigorous obesity classification in studies with small sample sizes.

### Measurement process and method

After selection of the suitable and hypothesis-relevant obesity measure, it is the researchers’ responsibility to ensure that the measurement guidelines are thoroughly followed to reduce measurement error. For instance, WC is widely accepted as a simple and reliable measure of obesity in general, and abdominal obesity, in particular. There exist guidelines to ensure WC is appropriately measured [29, 30]. However, Agarwal et al. [31] found significant differences in the measured WC across varying anatomical sites, phases of respiration, and time since last meal, when following either the WHO or the National Institute of Health (NIH) guidelines. Overlooking these details can lead to an increase in the measurement error and the steps taken to control them should be acknowledged in publications.

Similarly, the cut-off values used to classify subjects into distinct risk groups are also worth scrutiny. For instance, WHO identifies 25 and 30 kg/m^2^ as BMI cut points for overweight and obesity respectively. However, it has been shown that among certain populations (e.g., individuals of Asian descent) cardio-metabolic risk is increased at lower body mass indexes [32]. While some researchers advocate using international cut-offs [33], some find nationally and ethnically selected cut points, when available, more advantageous [34]. Overall, inconsistency in the cut-offs used across studies with similar topics is detrimental to the strength of the body of evidence.

This study aims to examine the obesity-related research in occupational health and safety regarding obesity measurement methods. The findings will show how researchers in the aforementioned fields are conducting obesity research and will inform future obesity research in the occupational safety and health domain.

## Methods

### Research strategy and study inclusion criteria

A systematic review of the PubMed database was undertaken with the following MeSH terms: (‘Work ‘or ‘Ergonomics’ or ‘Biomechanics\Biomechanical’ or ‘Occupational’ or ‘Motion’ or ‘Movement’) and (‘Obesity’ or ‘Skinfold Thickness’). In addition, a keyword search using Google Scholar and manual search of citations from relevant papers and literature reviews was done. The search was limited to journal articles dated between January 1, 2000 and December 31, 2014, published in English and studied human adults. An initial search was performed on 3 March 2015, and repeated on 24 September 2015 to update the search and results.

The study inclusion criteria were: (a) publication contributed to occupational health and safety rather than health promotion and (b) weight status was the independent variable or the major covariate included in the analysis and not the dependent variable. Review papers, simulation-based studies [35], and studies including normal weight subjects loaded with excess weight [36] were also excluded.

**Fig. 1:**
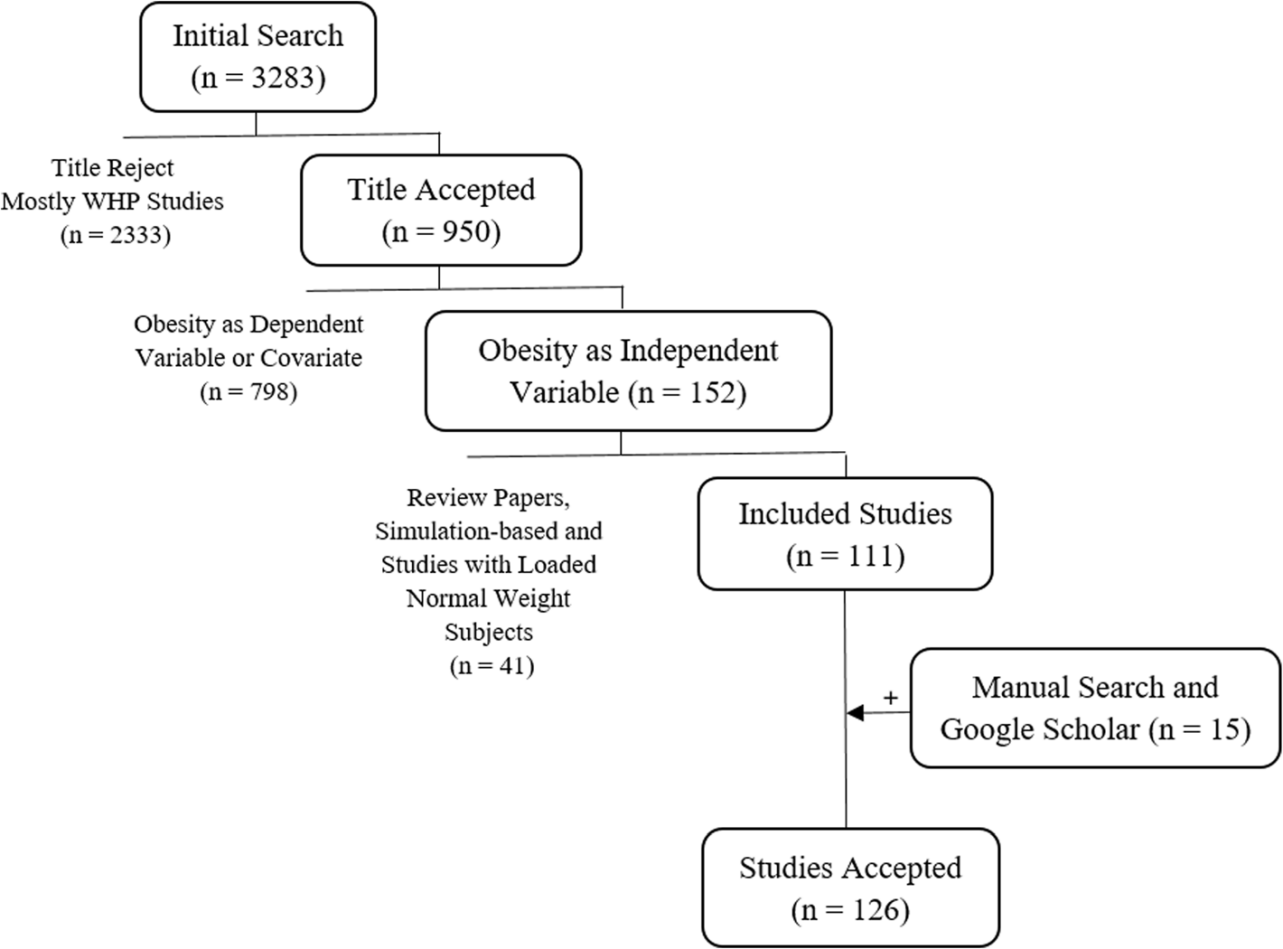
Flow Diagram of Study Selection. WHP indicates studies of Worksite Health Promotion that have addressed obesity. Search was also limited to studies that aimed to assess the effect of obesity on occupational or general physical functioning, rather than work-related risk factors for obesity. Studies using simulation software and loaded normal weight subjects were also excluded

The initial search resulted in 3283 studies. The first author assessed all search outcomes by title and/or abstract, out of which 950 were selected based on relevance of the topic. A review of the abstracts reduced the number of studies to 111. Manual searches of the references from these studies and Google Scholar added 15 studies that were not initially captured. Overall, 126 studies were selected (see Fig. 1).

### Selected studies review process and data extraction

Selected articles were reviewed and the following information was extracted: publication year, country of origin (based on the first author), main research question, design, outcomes measures, subject population, primary method of obesity measurement and the corresponding cut-offs used, whether or not subjects’ weight status was self-reported, additional obesity classification methods applied, the statistical method used, sample size and number of subjects in overweight/obese (OW/OB) subgroup, whether or not the study included women in the sample, the main finding and if the results indicated significance of the obesity (and overweight if applicable) effect, and finally if the authors mentioned any potential limitations of the obesity measurement method they have used. For studies carried out in a controlled lab setting where normal weight and overweight/obese subjects were compared, the mean BMI (or any other primary obesity classification measurement) of the overweight/obese group was extracted.

### Analysis

The main research question was categorized into two types: 1) general physical or mental work-related functioning (GF) and 2) task or body part specific functioning (TBS). The summary of all included studies, together with details about the study relevant to obesity classification are presented in Tables 1 and 2.

**Table 1:** The 37 studies that explored effects of obesity on general job-related outcomes, ordered chronologically

	Author, Year, Origin	Study Focus	Study Design	Subjects	Sample size (%OW/OB)	Outcome Variable(s)	Primary Obesity Measure / Other Measure(s)	Significant Obesity Effect	Significant Overweight Effect	Acknowledging Limitations of Obesity Measures
General physical or mental work-related functioning	Lee et al.,2001, Australia [48]	Asbestos exposure	Secondary data analysis	Former Australian mine workers	693 (68%)	Pleural thickening	**BMI**	+	–	+
	Clark et al.,2002, USA [49]	Duty fitness	Cross-sectional	Active firefighters(white)	218 (81%)	EKG, VO2 max, METS	**BMI**	+	+	+
	Poston et al.,2002, USA [50]	Discharge from training	Prospective cohort	Airmen	32,144 (19%)	Discharge status	*BMI	–	+	+
	Arbabi et al.,2003, USA [51]	Crash injury patterns	Secondary data analysis	Hospital admits of car crash	189 (57%)	Injury Scale and max AIS score, injury severity	BMI	+	–	+
	Bungum et al.,2003, USA [52]	Healthcare costs, absenteeism	Cross-sectional	Permanent employees	506 (74%)	Annual healthcare cost, absent days	BMI	+	NA	–
	Moreau et al.,2004, Belgium [53]	Sick leave	Prospective cohort	Belgium workers	20,463 (57%)	Sick leave	BMI / WC	+	+	–
	Pronk et al.,2004, USA [54]	Work performance	Cross-sectional	Current active employees	683 (43%)	# of work loss days, job performance, extra effort exerted, interpersonal relationships	**BMI**	+	–	–
	Laitinen et al.,2005, Finland [55]	Working ability	Prospective cohort	Young adults	11,637 (19%)	Perceived work ability	BMI / WHR	+	+	+
	Ricci & Chee,2005, USA [56]	Lost productive time	Cross-sectional	Employed adults	7472 (58%)	Self-reported lost productive time in past 2 weeks, lost labor costs	BMI	+	–	+
	Arena et al.,2006, USA [57]	Short-term disability	**Retrospective cohort**	White collar employees	1690 (37%)	Frequency + duration of short term disability	BMI	+	+	–
	Cormier & Israel-Assayag,2006, Canada [58]	Inflammatory response	Retrospective + experimental	Pig farmers + general population	14 (57%)	Inflammation biomarkers: C-reactive protein, interleukin 6,soluble adhesion molecules,	BMI / *Girth Size	NA	+	–
	Nishitani & Sakakibara,2006, Japan [59]	Job stress	Cross-sectional	Japanese manufacturing workers	208 (32%)	Job characteristics, eating behavior,	**BMI**	+	NA	–
	Wang et al.,2006, USA [60]	Healthcare costs	**Cross-sectional**	Manufacturing company employee & spouses	35,932 (74%)	Medical and pharmaceutical claims	BMI	+	+	–
	Østbye et al.,2007, USA [61]	Compensation claims, costs, lost workdays	Retrospective cohort	Health care and university employees	11,728 (56%)	Workers’ compensation claims, associated costs, and lost workdays	BMI	+	+	–
	Charles et al.,2007, USA [62]	Hemato-logic parameters	**Cross-sectional**	Police officers	104 (78%)	White blood cell and platelet counts	BMI / WC,WHR, hip circumference, abdominal height, waist to height ratio	+	+	–
	Finkelstein et al.,2007, USA [63]	Injuries/ treatment costs	Cross-sectional	General population	42,304 (62%)	Medically attended injury rates by mechanism and nature and related treatment costs	BMI	+	+	–
	Jans et al.,2007, Netherlands [64]	Absenteeism	Prospective cohort	Employees in industrial, administrative, and service sectors	1284 (40%)	Company-reported absenteeism	BMI	+	–	–
	Gates et al.,2008, USA [65]	Presenteeism	Cross-sectional	Manufacturing company employees	341 (78%)	Work Limitations Questionnaire	BMI	+	–	–
	Soteriades et al.,2008, USA [66]	Job disability	Prospective cohort	Firefighters	329 (88%)	Job disability	BMI	+	–	+
	Claessen et al.,2009, Germany [67]	Work disability	**Prospective cohort**	Construction workers	16,875 (63%)	# of cases	BMI	+	–	–
	Vissers et al.,2009, Belgium [68]	Whole body vibration	Lab-based	**Premenopausal women**	20 (100%)	Ventilation of oxygen, carbon dioxide, heart rate	*BMI / %BF: skinfold thickness	+	+	+
	Bedno et al.,2010, USA [69]	Heat illness/healthcare utilization	Prospective cohort	Active duty US army members	9667 (57%)	Heat illness incidence	weight for height / %BF, BMI	+	–	–
	Robroek et al.,2010, Netherlands [70]	Productivity loss /sick leave	Cross-sectional	Workers	10,624 (49%)	Sick leave, self-reported productivity loss	BMI	+	–	–
	Vincent et al.,2010, USA [71]	Fear of movement	Cross-sectional	Patients with knee pain diagnoses	278 (73%)	Fear of movement, knee function	*BMI	+ (only morbid obesity)	–	–
	Cowan et al.,2011, USA [72]	Training-related overuse injuries	Cross-sectional	Active duty US army members	7323 (47%)	Musculoskeletal injuries incidence and healthcare utilization	Weight for height / %BF, BMI	+	NA	–
	Poston et al.,2011, USA [73]	Absenteeism	Cross-sectional	Career firefighters	478 (19%)	Injury, and injury-related absenteeism	BMI / %BF: BIA, WC	+	+	–
	Haukka et al.,2012, Finland [74]	Multisite musculoskeletal pain	Prospective cohort	**Kitchen workers**	385 (46%)	Multisite musculoskeletal pain (3 and above out of 7)	BMI	+	–	–
	Caberlon et al.,2013,Brazil [75]	Musculoskeletal pain	Cross-sectional	Obesity treatment patients	95 (100%)	Musculoskeletal symptoms	*BMI	+	NA	–
	Gubata et al.,2013, USA [76]	Mental disorders	**Prospective cohort**	Active duty US army members	11,369 (40%)	Onset of mental disorder	*Circumference taping / BMI, weight-for-height standard, %BF	–	–	–
	Jahnke et al.,2013, USA [77]	(Musculoskeletal) injury	Prospective cohort	Firefighters	301 (0%)	Incident injury, MS injury	BMI / %BF: BIA, WC	+	+	–
	Kouvonen et al.,2013, UK [78]	Occupational injury	Prospective cohort	Finnish hospital workers	69,515 (0%)	Occupational injury incident	BMI	+	+	–
	Lin et al.,2013, USA [79]	Occupational injury	Prospective cohort	Civilian labor force	~ 7000 (50%)	Injury at work	BMI	+	–	+
	Roos et al.,2013, Finland [80]	Disability retirement	Prospective cohort	Middle aged employees	6542 (50%)	Pensions register data & questionnaire	BMI	+	NA	–
	Van der Starre et al.,2013, Netherlands [81]	Need for recovery	Cross-sectional	Office workers	412 (42%)	Need for recovery after work	BMI	+	–	+
	Viester et al.,2013, Netherlands [82]	Musculoskeletal symptoms/recovery	Cross-sectional + longitudinal	Dutch workforce	44,793, 2nd phase: 7909, (43%)	Musculoskeletal symptoms	BMI	+	+/−	+
	Gonzales et al.,2014, USA [83]	Cognitive functionality	**Lab-based**	General population	73 (67%)	Blood oxygen level-dependent response	WC / BMI	+	NA	–
	Smith et al.,2014, USA [84]	Mental disorders	Secondary data analysis	Military personnel	15,195 (61%)	Mental health disorders	BMI	+	+	–

For primary obesity measure, * indicates that the study reported mean of obesity measure for obese group. Bolded measure indicates that a cut-off other than the common cut-offs are used and underlined measure indicates that measurement has been based on self-reported data. For study subjects, bolded indicates that only females were included as subjects and underlined shows that males were the only subjects. A bolded study design indicates that obesity status had been considered as continuous variable while underlined bolded indicates that it had been considered both as a continuous and categorical variable

**Table 2:** The 89 studies that explored effects of obesity on task or body part specific functioning, are categorized into 7 groups based on their main focus and ordered chronologically within groups

	Author, Year, Origin	Study Focus	Subjects	Sample size (%OW/OB)	Outcome Variable(s)/Method	Primary Obesity Measure / Other Measure(s)	OB BMI: mean(SD)/ range	Significant Obesity Effect	Significant Overweight Effect	Acknowledging Limitations of Obesity Measures
Gait Characteristics	DeVita et al., 2003, USA [85]	Lower extremity joint kinetics & energetics	General population	39 (54%)	Motion analysis, force platform	BMI	42.3(2.9)	+	NA	–
	Browning et al., 2006, USA [86]	Metabolic rates & energy cost	General population	39 (49%)	Oxygen consumption, preferred walking speed	BMI / WHR, %BF: DEXA	M:33(2).1 F:33.8(3.3)	+	NA	–
	Browning & Kram., 2007, USA [87]	Walking biomechanics(knee-joint loads)	Young adults	20 (50%)	Ground reaction force, gait kinematics	BMI / *segment mass	M:34.1(3.7), F: 37(6)	+	NA	–
	Lafortuna et al., 2008, Italy [88]	Energetics and cardiovascular responses of walking & cycling	**Lean: hospital staff, OB: hospital admits (body mass reduction)**	21 (71%)	**HR, Vo2 max, metabolic rate**	BMI / %BF: BIA	41.1(5)	+	NA	–
	Lai et al., 2008, China [89]	Three-dimensional gait characteristics	General population	28 (50%)	Motion analysis	**BMI**	33.06(4.2)	+	NA	–
	Browning et al., 2009, USA [90]	External mechanical work	Young adults	20 (50%)	Ground reaction force	BMI	M:34.1(3.7), F:37(6)	–	NA	+
	Malatesta et al., 2009, Switzerland [91]	Mechanical external work	General population	49 (61%)	**Center of mass displacement, mechanical external work, kinetic energy transduction**	BMI	39.6(0.6)	–	NA	–
	Ko et al., 2010, USA [92]	Characteristics of gait	Older adults enrolled in aging research	164 (66%)	Motion analysis, force platform	BMI		+	+/−	–
	Russell et al., 2010, USA [93]	Energy expenditure & biomechanical risk factors for knee OA	**Young adults**	20 (50%)	O2 uptake, peak impact shock, peak external knee adduction moment knee adduction angular impulse	BMI	33.09(4.22)	–	NA	–
	Blaszczyk et al., 2011, Poland [94]	Basic spatiotemporal gait measures	**General population + outpatient obesity treatment clinic**	136 (74%)	**Stance & swing time, stride length**	BMI	37.2(5.2)	+	NA	+
	Ehlen et al., 2011, US [95]	Energetics and biomechanics of gait	General population	12 (100%)	Oxygen consumption, ground reaction forces, & three-dimensional lower-extremity kinematics	BMI / %BF: DEXA	33.4(2.4)	NA	NA	–
	Cimolin et al., 2011, Italy [96]	Gait pattern	**Obese: admits to obesity multidisciplinary rehabilitation program**	28 (64%)	Gait Spatio-temporal parameters & kinematics	BMI / WC	OB + LBP: 42.4(5.5), OB - LBP: 39.3	+	NA	–
	Russell & Hamill., 2011, US [97]	Obesity × laterally wedged insole effect on gait kinetic and kinematic	**Young females**	28 (50%)	Peak joint angles, external knee adduction moment & angular impulse	BMI / %BF: DEXA	37.2(6.1)	+	NA	–
	Wu et al., 2012, USA [98]	Gait adaptations & implication on risk of slip initiations	Young male students	10 (50%)	Motion analysis, force plate	%BF from BIA / BMI	33.7(2.8)	+/−	NA	–
	Harding et al., 2012, Canada [99]	Knee OA × obesity effect on knee joint mechanics	General population + orthopedic clinic admits	244 (72%)	Knee joint angles, joint moment	BMI / *thigh and calf circumference	34.9(4)	+	+	+
	Russell et al., 2013, USA [100]	Laterally wedged insoles × obesity effect on knee joint contact force	**General population**	28 (50%)	Center of pressure on the tibial plateau	BMI / %BF: DEXA	37.2(6.1)	NA	NA	–
	Browning et al., 2013, USA [101]	Metabolic rate, stride kinematics & external mechanical work	**young females**	37 (49%)	Oxygen uptake, ground reaction force, lower extremity kinematics	BMI / %BF: DEXA, *Trunk-to-leg fat mass ratio	33.9(3.6)	–	NA	–
	Ranavolo et al., 2013, Italy [102]	Walking coordination during walking	General population	50 (50%)	**Motion analysis**	BMI / WC, %BF: Siri equation	Range(33.8–44)	+	NA	–
	Vismara et al., 2014, Italy [103]	Changes in gait	General population	32 (44%)	Motion analysis	BMI	40.2(3.3)	+	NA	–
	Haight et al., 2014, USA [104]	Compressive tibio-femoral forces	General population	19 (47%)	Motion analysis (lower extremity biomechanics), EMG	BMI / %BF: DEXA	35(3.8)	+/−	NA	–
	Glave et al., 2014, USA [105]	Gait alterations	**General population**	22 (50%)	Gait variables	BMI / %BF: DEXA	31.42(7.3)	+	NA	+
	Cau et al., 2014, Italy [106]	Gait strategy	Hospital patients for weight reduction programs & staff	35 (57%)	Center of pressure parameters	BMI	43(4.9)	+	NA	–
	Lerner et al., 2014, USA [107]	Joint kinematics & individual muscle forces during gait	General population	19 (47%)	Motion analysis, EMG data, ground reaction force	BMI / lean mass (kg): DEXA	35(3.78)	+	NA	–
Disease Prevalence/Incidenc	Kouyoumdjian et al., 2000, Brazil [108]	Severity of Carpal tunnel syndrome	Carpel tunnel syndrome patients	384 (13%)	Case - control study	BMI		+	NA	–
	Young et al., 2001, USA [109]	Asthma risk	Military population and their families (17-69 yrs)	38,924 (53%)	**Case - control study**	BMI		+	+	–
	J. D. Bland., 2005, UK [110]	Age × body mass index effect on carpel tunnel syndrome risk	Hospital admits	4166 (14%)	**Self-report CTS diagnosis**	BMI		+	+	–
	Liuke et al., 2005, Finland [111]	Prevalence and progression of lumbar disc degeneration	Employed middle-aged men	129 (50%)	Prospective cohort: MRI imaging	BMI		NA	+	–
	Dagan et al., 2006, Israel [112]	BMI as a screening method for detection of excessive daytime sleepiness	Professional drivers	153 (100%)	**Sleep characteristics**	BMI	36.78(7.32)	+	NA	–
	Zhao et al., 2007, USA [113]	Osteoporosis	Chinese general population + US Caucasian general population	6477 (0%)	**Bone mass at the lumbar spine, total body bone mineral content**	BMI / %BF: DEXA		+	+	+
	Sharifi-Mollayousefi et al., 2008, Iran [114]	BMI as independent risk determinants in the development and severity of Carpal tunnel syndrome	Patients with carpal tunnel syndrome (cases) and their relatives (controls)	262 (50%)	**Case-control study**	BMI		+	NA	–
	Grotle et al., 2008, Norway [115]	OA incident in hip, knee, and hand	General population	1675 (35%)	Prospective cohort: OA diagnosis	BMI		+	+/−	–
	Noorloos et al., 2008, Netherlands [116]	Obesity × whole body vibration effect on risk of LBP	Occupational vehicle drivers	214 (69%)	**Low back pain**	BMI		–	–	–
	Toivanen et al., 2010, Finland [117]	Knee OA risk	Finnish adults aged 530 years	823 (39%)	Prospective cohort: OA diagnosis	BMI		+	+	–
	Vismara et al., 2010, Italy [118]	LBP incidence	**General population**	37 (70%)	Trunk angle during standing, forward flexion & lateral bending	BMI	LBP:41.9(5.3),Non:39.2(3.6)	+	NA	–
	Wood et al., 2011, USA [119]	Pain experienced by persons with chronic back pain	Patients with lower back pain of over 3 months	198 (62%)	**Blood pressure, pain level**	BMI		–	–	–
	Ackerman & Osborne., 2012, Australia [120]	Burden of hip & knee joint disease	General population	1157 (55%)	OA diagnosis	BMI		+	+	–
	Jensen et al., 2012, Denmark [121]	LBP risk factor	**Newly educated health care helpers**	1355 (41%)	Prospective cohort: Self-reported levels of LBP	BMI	34.8(6.08)	–	–	–
	Silvernail et al., 2013, USA [122]	Biomechanical risk factor for knee OA	Yong university and community members	30 (67%)	Gait kinetic & kinematics	BMI / %BF: BIA	34.4(3.9)	–	–	–
	Seror & Seror., 2013, France [123]	Incidence of idiopathic median nerve lesion at the wrist	Patients with carpal tunnel syndrome	676 (25%)	Electrophysiological evaluation outcomes	BMI		+	+	–
	Martin et al., 2013, USA [124]	Knee OA risk factor	British birth cohort participants	2957 (0%)	Knee Osteoarthritis	BMI (z-score)		+	NA	–
	Romero-Vargas et al., 2013, Mexico [125]	Modifications on spino-pelvic parameters & type of lumbar lordosis	General population	200 (80%)	**Spino-pelvic values**	BMI / WC		–	–	+
	Messier et al., 2014, USA [126]	Frontal plane knee alignment × obesity effect on knee joint loads in knee OA	Community dwelling older adults (age > 55 yrs)	157 (100%)	knee osteoarthritis: X-ray at baseline	BMI	33.4(3.7)	+	+	–
	Urquhart et al., 2014, Australia [127]	Occupational activities × obesity effect on LBP	**General population + weight loss clinic attendees**	145 (61%)	Low back pain intensity & disability	BMI		+	NA	–
	Evanoff et al., 2014, France [128]	Physical occupational exposures × obesity effect on post-retirement shoulder/knee pain	French national power utility employees	9415 (52%)	Retrospective cohort: self-administered questionnaires	BMI		+	–	–
Functional Capacity	Hulens et al., 2001, Belgium [129]	Submaximal & maximal exercise capacity	**General population**	306 (74%)	**Oxygen uptake, carbon dioxide production, respiratory quotient, breathing efficiency, mechanical efficiency & anaerobic threshold**	**BMI** / %BF: BIA	38.1(5.6)	+	NA	–
	Hulens et al., 2002, Belgium [130]	Peripheral muscle strength	**Outpatient Endocrinology Clinic patients**	241 (100%)	**Trunk strength, peak oxygen consumption**	BMI / Fat free and fat mass: BIA	37.5(5.4)	NA	NA	–
	Maffiuletti et al., 2007,Switzerland [131]	Voluntary & stimulated fatigue of the quadriceps femoris muscle	Lean: hospital staff, obese: hospital admits for body mass reduction	20 (50%)	Maximal voluntary isometric & isokinetic torque, torque loss	BMI / Fat free mass: BIA	41.3(5.4)	+	NA	–
	Segal et al., 2009, USA [132]	Forces on the medial compartment of the knee joint	General population	59 (68%)	**knee joint forces**	BMI / WHR	Central: 35(4), lower body: 36.4 (5.4)	+/−	NA	+
	Capodaglio et al., 2009, Italy [133]	Lower limb muscle function	General population	40 (50%)	Isokinetic strength during knee flexion & extension	BMI	38.1(3.1)	+	NA	+
	Singh et al., 2009, USA [134]	Maximum acceptable weights of lift	General population	60 (67%)	**MAWL**	BMI / WC,WHR,%BF estimated: ST	II: 37.13(1.58) III:47.84(9.85)	–	NA	–
	Faria et al., 2009, Portugal [135]	Muscle–tendon unit stiffness	**General population**	105 (77%)	Ankle muscle–tendon unit stiffness at 30% MVC	BMI	32.1(1.3)	+	+	–
	Park et al., 2010, USA [136]	Joint RoM	Young and university affiliated	40 (50%)	RoM	BMI	44(7.4)	+/−	NA	–
	Blazek et al., 2013, USA [137]	Age × obesity effect on Knee adduction and flexion moments	General population	96 (38%)	Ground reaction force magnitude, knee alignment, step width, toe-out angle, limb position	BMI	35.3(3.9)	+	NA	–
	Cavuoto & Nussbaum., 2013, USA [138]	Age × obesity effect on shoulder capacity	Young: students, old: retired or employed in non-physically demanding jobs	32 (50%)	Endurance, discomfort, motor control, task performance	BMI / WC, WHR	Young: 34.1(2.8), Old: 36.4(3.3)	+	NA	+
	Hamilton et al., 2013, USA [139]	BMI × workstation configuration effect on joint angles	General population	30 (80%)	Joint angle, forward functional reach	**BMI**	I: 32(1.26) II:37(1.73) III:44(4.97)	–	–	–
	Mignardot et al., 2013, France [140]	Motor control behavior	General population	20 (60%)	Kinematic variables, Center of mass displacement characteristics	BMI	36.6(3.3)	+	NA	–
	Wearing et al., 2013, Australia [141]	Resistance exercise × obesity effect on immediate transverse strain of the Achilles tendon	University faculty	20 (50%)	Sonographic examinations	**BMI**	30(3.1)	+	+	+
	Cavuoto & Nussbaum., 2013, USA [142]	Strength and functional performance	Local community	36 (50%)	Endurance time, strength	BMI / WC,WHR	33.6(3.1)	+/−	NA	+
	Cavuoto & Nussbaum., 2014, USA [143]	Age × obesity effect on functional performance	General population	32 (50%)	endurance, discomfort, motor control, task performance	BMI / WC,WHR	Young: 34.3(4), Old: 35.9(3.6)	+	NA	+
	Mehta & Cavuoto., 2015, USA [144]	Obesity × age effects on handgrip endurance	**General population**	45 (44%)	hand grip endurance	BMI	Young: 33.1(3.6),Old:36.1(8.1)	+/−	NA	–
Balance & Plantar Pressure	Hills et al., 2001, Australia [145]	Plantar pressure	General population	70 (50%)	**Pressure distribution**	BMI	38.75(5.97)	+	NA	+
	Gravante et al., 2003, Italy [146]	Centre of pressure location & plantar pressures	General population	72 (53%)	Centre of pressure location, plantar ground contact surface areas & pressures	BMI / WHR	M:36(7.4), F:38(6.8)	+/−	NA	–
	Birtane & Tuna., 2004, Turkey [147]	Plantar pressure distribution	General population	50 (50%)	**Pedobarographic evaluations**	BMI	32.2(2)	+	NA	–
	Berrigan et al., 2006, Canada [148]	Balance control constraint during accurate and rapid arm movement	General population	17 (53%)	Body kinematics, center of pressure, displacement, reaction time, movement time	**BMI**	37(6.6)	+	NA	–
	Teh et al., 2006, Singapore [149]	Pressure distribution under the feet	General population	120 (42%)	Plantar pressure distribution	BMI	I: 34.3 II: 38.9(3.6)	+/−	NA	–
	Singh et al., 2009, USA [150]	obesity × task duration effect on postural sway and functional reach	Students & sedentary office workers	20 (50%)	Posture sway, functional reach	BMI / WHR	45.96(7.85)	+	NA	–
	Blaszczyk et al., 2009, Poland [151]	Postural control	**Obesity treatment clinic patients**	133 (75%)	**CP measures: voluntary displacement, path, range**	BMI / %BF: BIA, WC,HC	37.2(5.2)	–	NA	–
	Park et al., 2009, USA [152]	Postural stress during static posture maintenance	General population	40 (50%)	Rated perceived exertion	BMI / WHR,%BF estimated: ST	46.26(4.99)	+	NA	–
	Menegoni et al., 2009, Italy [153]	Static posture variability	Orthopedic Rehabilitation Unit patients and staff (control)	54 (81%	**Center of pressure velocity & displacements along the antero-posterior & medio-lateral axis**	BMI	M:40.2(5), F: 41.1(4.1)	**+**	NA	**+**
	Monteiro et al., 2010, Portugal [154]	Plantar pressure	**Postmenopausal women**	239 (79%)	Foot-scan pressure plate	%BF from BIA / **BMI**	29.6(3.2), 36.4(3.8)	+/−	NA	**–**
	Miller et al., 2011, USA [155]	Balance recovery from small forward postural perturbations	Young adults (22 years old)	20 (50%)	Peak COM displacement, peak COM velocity, peak ankle torque	BMI	33.2(2.3)	–	NA	–
	Matrangola & Madigan., 2011, USA [156]	Balance recovery using an ankle strategy	Young males	20 (50%	Body angle, ground reaction force	BMI	32.2(2.2)	+/−	NA	–
	Peduzzi de Castro et al., 2014, Portugal [157]	Pressure relief insoles	General population	31 (32%)	Ground reaction force, plantar pressure	BMI	36.5(4.51)	+	NA	–
Task Functionality	Galli et al., 2000, Italy [158]	Motion strategies: sit-to-stand	General population + obese subjects suffering from chronic lower back pain	40 (75%)	Movement kinetics & kinematics	BMI	40(5.9)	+	NA	–
	Sibella et al., 2003, Italy [159]	Biomechanical model: sit-to-stand	Hospital recovers	50 (80%)	Trunk flexion, feet movement, knee & hip joint torques	BMI	37.9(4.9)	+	NA	–
	Lafortuna et al., 2006, Italy [160]	Energy cost of submaximal cycling	**Lean: hospital staff, obese: hospital admits for body mass reduction**	18 (50%)	**Oxygen uptake, Vo2 max, anaerobic threshold, mechanical efficiency**	BMI / %BF: BIA	40(1.2)	+	NA	–
	Gilleard & Smith., 2007, Australia [161]	Postural adaptations: trunk forward flexion motion in sitting and standing	**General Population**	20 (50%)	**Trunk flexion motion during forward flexion, trunk posture, hip joint moment**	WC / BMI	38.9(6.6)	+/−	NA	–
	Xu et al., 2008, USA [162]	Lifting kinematics & kinetics	College students	12 (50%)	Motion analysis	BMI	33.28 (30.4–38.8)	–	NA	+
	Taboga et al., 2012, Italy [163]	Mechanical work, energy cost of transport, and efficiency: running	Hospital admits-adults from metabolic disorders	25 (40%)	Oxygen uptake, kinematics, center of mass location	BMI / %BF: BIA	41.5(5.3)	+	NA	–
	Hendrick et al., 2012, USA [164]	Neural processes of cognitive control: stop signal test	**General population**	43 (30%)	**Functional magnetic resonance imaging**	**BMI**	33.2(2.6)	+	NA	+
	Singh et al., 2013, USA [165]	Contact forces & moments exerted by the abdomen on the thigh: seated reaching	Older adults	10 (100%)	**Motion analysis, force plate**	BMI / WC	39.04(5.02)	+/−	NA	+
	Schmid et al., 2013, Switzerland [166]	Kinetic & kinematic variables: sit-to-stand test.	**Going to attend a weight loss program at hospital**	36 (72%)	Vertical ground reaction forces, rising velocity (motion analysis)	BMI	I: 32.68(1.53), II: 39.42(2.71)	–	+/−	–
Pysiological Responses	Willenberg et al., 2010, Switzerland [167]	Venous flow parameters of the lower limbs	Students and medical staff	45 (49%)	**Venous hemodynamics: Diameter, flow volume, peak, mean, & minimum velocities**	BMI / WHR, WC	36.2(5.9)	+	NA	+
	Engelberger et al., 2014, Switzerland [168]	Diurnal leg volume increase	Obese subjects: weight management clinic patients, general population	39 (62%)	Common femoral vein diameter, peak flow velocity, mean velocity & minimal velocity	BMI / WHR	40.2(5.9)	+	NA	–
	Yang et al., 2015, China [169]	Acute high-altitude exposure	Chinese railroad construction workers	262 (46%)	Acute mountain sickness	**BMI**	29.9(3.8)	–	NA	–
Miscellaneous	Menegoni et al., 2007, Italy [170]	Clinical protocol to characterize the trunk movements	**Lean: hospital staff, obese: hospital admits for diet therapy and exercise classes**	20 (50%)		BMI	38.7(3.5)	NA	NA	–
	Forman et al., 2009, USA [171]	Restraint of automobile occupants	**Post mortem human surrogates**	5 (40%)	Chest deformation, acceleration, tension in the restraint system, etc.	BMI	40	+	NA	–
	Lerner et al., 2014, USA [172]	Obesity-specific kinematic marker set to account for subcutaneous adiposity	General population	18 (50%)	Ground reaction force, walking kinematics, EMG	BMI	35(3.78)	NA	NA	–
	Thorp et al., 2014, Australia [173]	Standing workstations effect on fatigue, musculoskeletal discomfort & work productivity	Middle-aged sedentary employees	23 (100%)	Self-reported fatigue, musculoskeletal discomfort, work productivity	BMI	33.7(4.3)	+	+	–

For primary obesity measure, Bolded measure indicates that a cut-off other than the common cut-offs are used and underlined measure indicates that measurement has been based on self-reported data. For study subjects, bolded indicates that only females were included as subjects and underlined shows that males were the only subjects. A bolded outcome variables/method indicates that obesity status had been considered as continuous variable while underlined bolded indicates that it had been considered both as a continuous and categorical variable

## Results

**Fig. 2:**
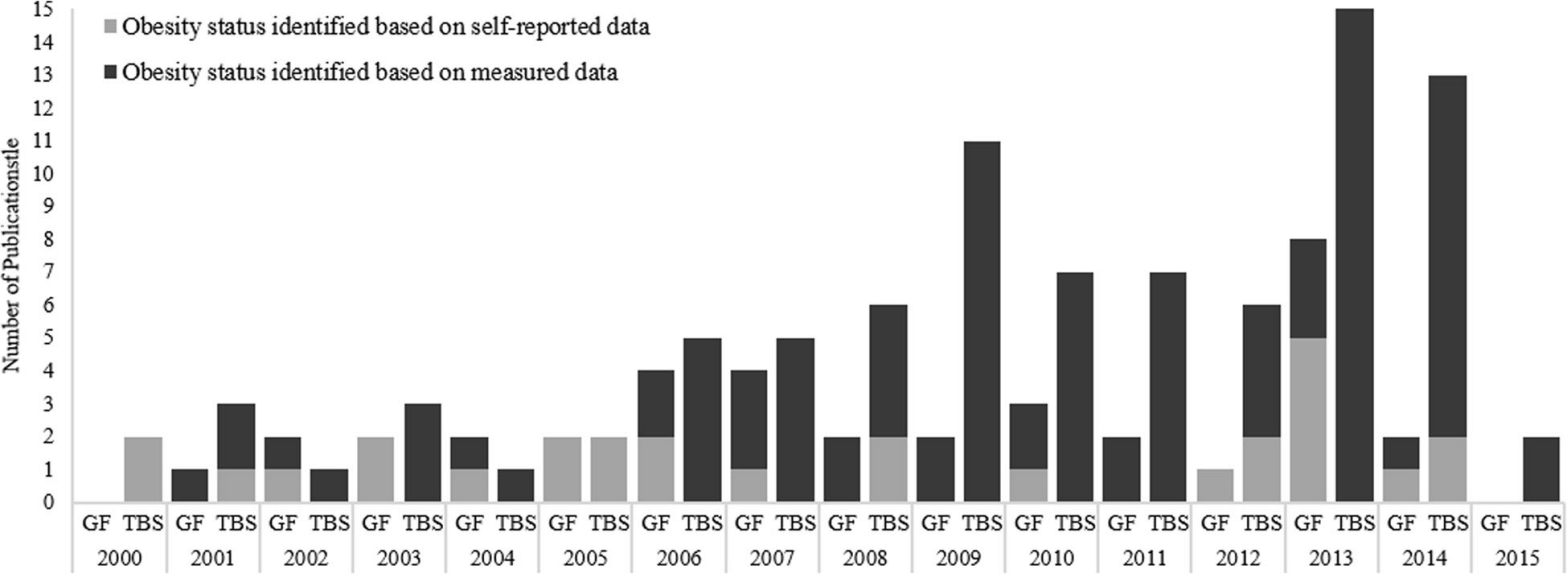
Over the period of the review, there was an increasing interest in obesity research

Within the selected time period (2000–2015), there has been an increasing trend in the number of studies published (see Fig. 2), with 2013 having the maximum number of publications (*n* = 23). This increase is mostly owed to the expanding interest in the specific effects of obesity rather than the general effects, which have been steadily studied by, on average, *n* = 2.4(SD = 1.9) studies per year. Overall, among all included studies, 63% relied solely on BMI to distinguish obese from non-obese and further classify them into distinct obesity status sub-groups (see Fig. 4). This is particularly concerning because some of these were lab-based studies with sample sizes as small as 12 or used young adults or older adults enrolled in aging research as subjects. In the following sections, studies focused on general vs. specific effects of obesity are discussed separately.

### Category 1: General physical or mental work-related functioning

**Fig. 3:**
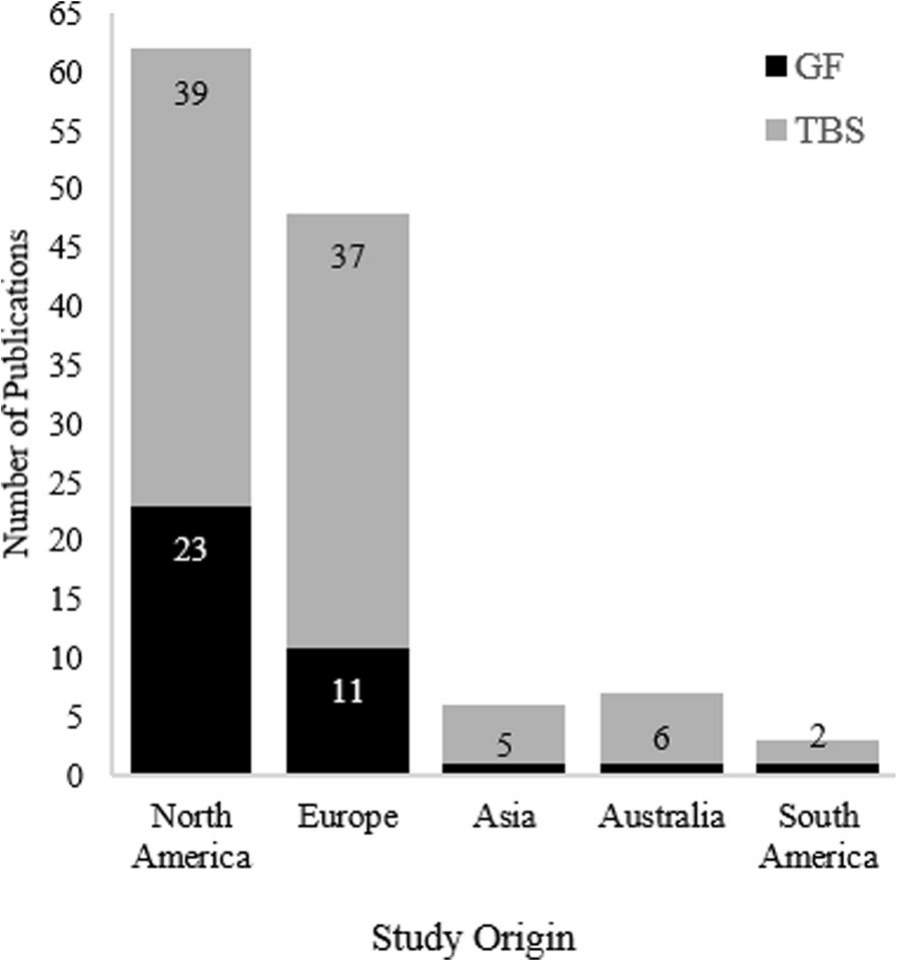
The origin of the studies. As expected, North America had the highest number of publications in both GF and TBS categories

Among the 126 reviewed studies, 37 were related to the general effects of obesity as they pertain to occupationally-relevant outcomes such as performance, disability and discharge rate, healthcare cost, and overall well-being (see Table 1). The majority (64%) of the studies were from North America (see Fig. 3). Over the period of the review, the topic of general studies has gradually moved from work performance and workplace costs associated with obesity to the potential reasons behind elevated costs and poor performance, such as musculoskeletal symptoms and mental health issues. These studies applied a wide range of designs, with cross-sectional being the most frequent (15), followed by longitudinal prospective studies (13). Participants in 10 studies were army personnel, police officers, or career firefighters and the rest were either civilian labor force (20) or their occupational status was not reported or relevant to the topic (7).

**Fig. 4:**
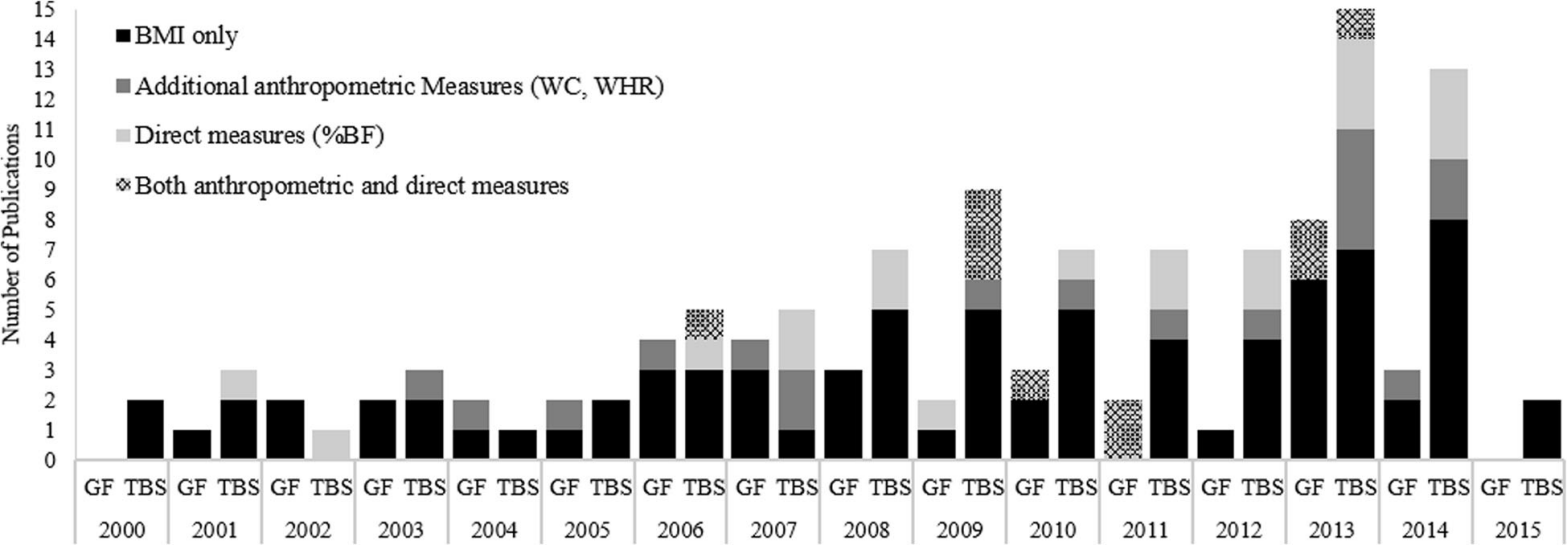
The obesity measurement methods used in studies. Regardless of the study category, BMI was the most frequently used method of obesity measurement

With regard to the measurement of obesity and group classification, in over 71% of these studies BMI was the only obesity measure used to distinguish obese from non-obese (see Fig. 4), with about 57% of these studies using self-reported weight and height to calculate BMI. About 13% of general studies used additional anthropometric measures such as WC and WHR to enhance obesity measurement accuracy. Finally, of the 6 studies using a direct adiposity measure, 5 were studies of army personnel, fire fighters, or police officers. Four studies reported using cut-off values other than 25 and 30 kg/m^2^ to categorize subjects into distinct BMI subgroups, out of which two were army studies, one included Asian participants, and one provided no justification to use BMI ≤ 26 kg/m^2^ as the cutoff for grouping. The median sample size was 1284 (14–69,515). With the exclusion of two studies that did not report the number of obese/overweight subjects included in their sample, on average 55.7(0.2) % of the samples consisted of OW/OB. Only 5 studies in this category provided information regarding the mean body mass index (or any other primary obesity classification measurement) of the OW/OB group. Overall, in 11 studies, the authors discussed the possibility of subject misclassification due to a reliance on BMI as the sole indicator of obesity status, either as a justification to use additional measures (2 studies) or as a limitation.

### Category 2: Task or body part specific functioning

The majority of the reviewed studies (89) investigated a wide range of specific effects of obesity (see Table 2). North America and Europe contributed by 45 and 39% of such studies, respectively (see Fig. 3). Authors from Italy in particular contributed 16% of the publications, ranking higher than Asia and Australia, with five and six studies respectively. It should be noted that region of origin did not systematically affect the measurement approach used. Although the majority of the studies in this category were laboratory-based observational studies, BMI was still the most frequently used measure, with 59% of the studies relying solely on it to distinguish obese from non-obese. Study topics varied broadly, however, they were categorized in seven groups based on their main hypothesis and research focus (shown in Table 2). These groups, ordered based on number of studies, are discussed in more details as follows.

**Fig. 5:**
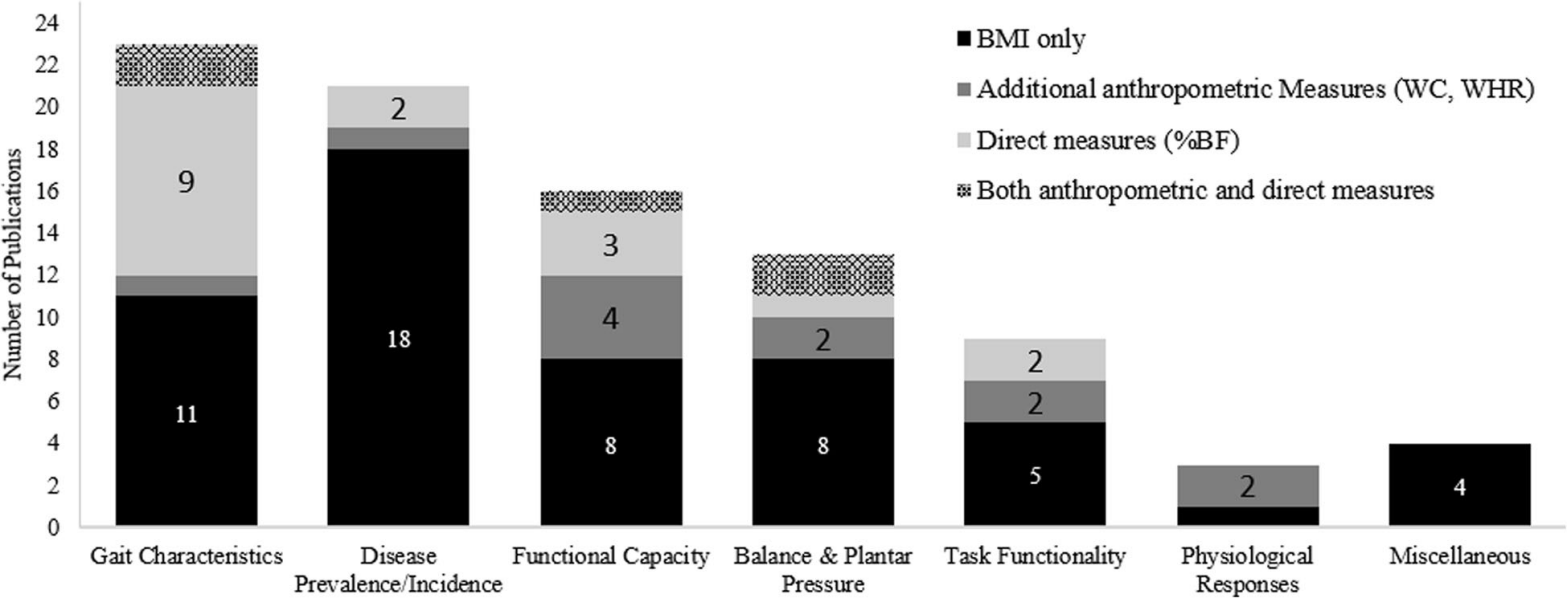
The obesity measurement methods used across the 7 sub-categories of studies that explored effects of obesity on task or body part specific functioning. With the exception of studies in gait categories, anthropometrics, in particular BMI, were still the most frequently used measures of obesity

Twenty-three studies (~ 26%) discussed how obesity alters outcomes related to gait, such as metabolic cost, preferred speed, spatio-temporal parameters, and joint moments. From 2006 to 2014, at least one study related to the effects of obesity on gait was published each year. All of the studies in the gait category were lab-based observational studies. While other studies recruited subjects from a general population, obese subjects in three European studies were females, sampled from obesity clinics. The median sample size was 28 (10–244). More than half of the included subjects (56.7%) were categorized as OW/OB (only two had an overweight group). There were 11 studies which used BMI only (see Fig. 5). With the exclusion of the studies which reported sex or condition-stratified averages (4), the average BMI for nine studies were ≤ 35 kg/m^2^, four were ≤ 40 kg/m^2^ and four were > 40 kg/m^2^. All but four studies reported a significant main effect for obesity or overweight on their outcomes of interest. It is noted that three of the studies reporting non-significant results used BMI as the sole obesity measure.

The next largest group focused on the prevalence, incidence, burden, and changes in symptoms of diseases such as carpel tunnel syndrome, osteoarthritis, low back pain (LBP), asthma, and sleep disorders in association with obesity. This category included some large scale public health studies, hence there was more diversity in terms of study design. The median sample size was 384 (30–38,924). With the exclusion of two studies that did not report the proportion of OW/OB, on average 54 (24) % of the samples were obese or overweight. Six studies used patients and hospital admits as participants and six studies reported subjects belonging to a certain occupation. Eighteen studies relied solely on BMI, two added %BF and one added WC. Sixteen studies failed to report the obesity class of the obese group. In the four that did, all but one had mean BMI ≤ 35 kg/m^2^. Only two studies, which both had one additional obesity measure, mentioned the inadequacy of BMI.

Changes in functional capacity were the topic of 16 studies. Functional capacity encompasses all topics related to muscle strength, endurance, functional reach, range of motion (RoM), and motor control behavior. Participants in two studies were outpatient clinic or hospital patients (endocrinology and body mass reduction admits) and the rest were recruited from the general population. Eight studies used BMI as the primary and only obesity measure, while three studies also measured body fat. Four studies augmented BMI with other anthropometric measures and one study reported using four obesity measures including both direct and indirect. While no studies relied on self-reported height and weight data, three studies used cut-offs other than 25 kg/m^2^ and 30 kg/m^2^ to classify subjects into distinct groups. Only three studies had an overweight sample as well as obese. The median sample size was 40 (20–306). On average ~ 60% of the sample were OW/OB and the majority of reported mean BMI values were in the range of 35–40 kg/m^2^. One Australian study in particular, which used BMI and cut-off values of 23 kg/m^2^ and 27.5 kg/m^2^, had mean BMI of 30 kg/m^2^ for obese. All but three studies reported some significant obesity effect and two reported significant overweight effect. Authors of six studies, out of which five had used multiple anthropometric measures, included a mention of BMI’s limitation as a measure of obesity.

Issues related to balance, postural stability, and plantar pressure were discussed by 13 lab-based studies. Subjects were recruited from the general population in all but three studies, two of which had sampled from orthopedic rehabilitation and obesity treatment clinic patients. In one study, %BF was the primary obesity measurement used, but eight studies used BMI as the primary and only measure. Two other studies used both %BF and other anthropometric measures, and two studies used both BMI and WHR. It is noted that the two studies with the largest sample sizes used %BF measured by BIA. One included obesity clinic patients and the subjects in the other were part of a health promotion program for postmenopausal women. The median sample size among studies using only BMI was 40.5 (17–120) out of which on average 51(14) % were obese. No study in this sub-category included overweight subjects. Two studies, one testing Canadian and one testing Portuguese subjects reported using BMI cut-offs other than 25 and 30 kg/m^2^. With the exception of three studies, the average BMI reported for subjects was above > 35 kg/m^2^ and in four studies the mean BMI was > 40 kg/m^2^. In terms of significance of the obesity effect, two study reported no significant effect and four reported some but not all outcome measures to be significantly affected by obesity. Only two studies had a mention of inadequacy of BMI, and they both used BMI only.

The effects of obesity on functionality while performing specific occupationally-relevant tasks was investigated by nine lab-based studies. All but one study tested physical tasks such as the sit-to-stand movement, lifting, seated reach, cycling, and running. The remaining study focused on cognitive control. Three studies published by Italian authors tested hospital admits or recovering patients for body mass reduction or metabolic disorders. One study from Switzerland also recruited from individuals who were going to participate in a weight loss program at a hospital. Five studies relied on BMI only, while two added %BF measured by BIA and two added WC. The average sample size was 28.2 (SD = 14.5) and 60% of the included sample were obese. Only one study had an overweight group as well as obese. With the exception of two studies, the reported mean BMI for obese group was > 35 kg/m^2^. Five studies observed a significant effect of obesity on the performance of the specific tasks tested, while four reported no or partial effect. Three authors discussed how BMI is not the ideal obesity measure although only one used WC in addition.

Three studies discussed changes in physiological responses by obesity and the topics of four studies were not closely pertinent to the above mentioned subgroups. Details of these studies were reported in Table 2.

## Discussion

Researchers worldwide have investigated the effect of obesity (sometimes including overweight) on a wide range of occupationally-relevant outcomes. Experts from diverse disciplines, including but not limited to, public health, medicine, health sciences and engineering, have contributed to our current understanding of the magnitude of an effect of obesity at work [37]. The diversity of scientific disciplines involved in obesity research has both advantages and disadvantages. It allows for more complex aspects of the obesity effect to be revealed by diverse methodologies. However, it increases the risk of misuse of methods due to unfamiliarity. In particular, the investigators’ understanding of obesity and the methods to measure it and classify individuals into distinct risk groups can affect the quality of the findings.

The present study focused primarily on examining the use of various obesity measurement methods and secondarily on sampling strategies. Two categories of publications were considered: those investigating the effect of obesity on occupational disease development or business outcomes and those studying how obesity alters task-level performance or functional capacity. As expected, studies in the first category had large sample sizes and were mostly public health studies, carried out by public health professionals. While the samples mostly consisted of participants from the general population or a certain occupation, the large sample sizes justified the use of BMI as the sole obesity measure in over 70% of these studies. It is noted that the vast majority of the publications in this category failed to report descriptive statistics regarding the obesity status of the obese group included in the sample. This could serve as a critical source of information for comparative analyses. The WHO expert consultation [38] suggests that wherever possible, researchers should use all BMI categories for reporting purposes, in order to facilitate international comparisons (i.e., 18.5, 20, 23, 25, 27.5, 30, 32.5 kg/m^2^, and in many populations, 35, 37.5, and 40 kg/m^2^).

Another issue with studies in the general category is in regard to abdominal obesity. It is often defined using waist circumference, especially in recent years, while waist-hip ratio was often used in earlier years. However, various cut points have been recommended over time, by different health organizations and across countries, and used across studies. Abdominal obesity is a major component of metabolic syndrome, a cluster of metabolic abnormalities that carry an increased risk of cardiovascular diseases and diabetes [39]. However, there is a subset of the obese population that are metabolically healthy and their inclusion in study samples can confound the results. Ortega et al., [40] studied a large cohort of 43,265 individuals and reported that when adjusting for fitness and other confounders, metabolically healthy but obese individuals had lower risk of all-cause mortality, non-fatal and fatal cardiovascular disease, and cancer mortality than their metabolically unhealthy obese counterparts. In their study, over 46% of the obese sample were metabolically healthy. From the reported exclusion criteria in the studies reported here, it cannot be decided whether obesity would have the same effects in the absence of other components of metabolic syndrome, particularly for outcomes such as healthcare cost, job disability, absenteeism, and presenteeism.

The studies in the second category focused on task-level performance or functional capacity. There are three main points of discussion identified for these studies: 1) selection of obesity measurement(s) (e.g. BMI, WC, %BF) and the corresponding cut-points to distinguish obese from non-obese, 2) the study participants, both in terms of sample size and the population targeted (e.g. young adults, certain occupation groups, hospital admits), and 3) measurement considerations (e.g. site of measurement for WC). While these factors are all individually important, their interaction may also present a challenge to studies. For instance, when using BMI in a study with a small sample size, recruiting only young adults may be more problematic [41, 42] than when a larger group of older adults are classified based on BMI.

In this category, BMI was still the most frequently used obesity classification measure. Overall, the selection of an obesity measure should depend on the hypothesized mechanism by which the obesity effect would manifest. While obesity presents by both changes in anthropometry and metabolic function, acknowledgement of the considered causal pathway is advantageous to study rigor. Also, while obesity morphology may not be as crucial to the outcomes in the studies of the previous category, it is highly relevant to the dependent variables investigated by the studies in this category. In particular, balance and gait parameters are likely to be affected by the distribution of weight in the body, therefore not only obesity status, but also fat distribution needs to be taken into account. BMI by itself fails to do so, however other anthropometric measures such as WC and WHR are able to distinguish central obesity from lower body and general obesity. Across the 36 studies in the two aforementioned sub-categories, only 10 studies used additional anthropometric measures.

Caution should be made in the use of BMI in studies with small samples that include young adults. Statistically significant age dependencies have been reported in the relation between %BF and BMI, such that older adults have higher %BF compared with younger adults with comparable BMIs [25]. WHO expert consultation acknowledges the issue by stating that most studies show the relation between BMI and %BF to be dependent on age and sex, and also different across ethnic groups. Experts affirmed that Asian populations have different associations between BMI and percentage of body fat than do Western populations [38], however, due to lack of comprehensive data from all Asians, they suggested retaining WHO BMI cut-off points as international classifications. Using ethnic-specific cut-offs may come at the expense of consistency among studies. As such, we observed two studies from Portugal in the balance sub-category that participants were recruited from the general population, one using a BMI cut-off of 25.5 kg/m^2^ and the other using 30 kg/m^2^to distinguish obese from non-obese. Arbitrary grouping of subjects, not backed up by ethnic or other expected underlying differences, as was the case in these two studies, should also be minimized. Overall, it is alarming that only 20% of the studies in this category acknowledged the aforementioned shortcomings of BMI as the obesity measure.

The majority of studies in this category (~ 80%) were observational studies. To isolate an obesity effect, and in contrast to the majority of studies in the first category, subjects were selected such that they were mostly otherwise healthy. The representativeness of this group and the extent to which the findings based from them can be generalized to the overall obese population is concerning. This exclusion of obese with comorbidities from the study samples in this category and their possible inclusion in samples of the first category may contribute to the higher proportion of publications in the first category to report a significant obesity effect in comparison to the second category.

Another issue with the sample representativeness is including only severe obesity (classes II (BMI 35–39.9 kg/m^2^) and III (BMI ≥ 40 kg/m^2^). While this practice may be statistically sound and increase the likelihood of capturing the obesity effect, it again limits the generalizability of the result. For instance, in the United States the prevalence of obesity is estimated to be over 35% but less than 15% of the obese population (~ 5% of the total population) are categorized as class II and less than 7% (~ 2.5%) as class III [43].

There are considerations for proper use of each measurement as well. WC for instance is shown to be significantly different across sites of measurement, postures, phases of respiration, and time since last meal [31]. By following the existing measurement guidelines [44] studies are less prone to error and consistency across subjects and studies is also warranted. Also, practices such as having a single trained staff doing all the measurements when possible, keeping the measurement conditions homogenous across all subjects and using multiple measurements are beneficial for internal validity and worthy of report in research manuscripts.

Overall, we assessed obesity research in the occupational health field and showcased the practices of obesity measurement since 2000. The present study has many strengths, but also some limitations. While obesity has become a global epidemic, this review was limited to PubMed database as well as Google Scholar journal articles available in English, primarily due to the authors’ time and language proficiency constraints. Also, studies related to health promotion at work were excluded [45, 46]. Health risk assessment is a common part of these programs in which obesity status is commonly assessed as a health risk, however the topic of these studies were beyond the scope of this review. Moreover, although the effect of certain work types, such as shift work on the onset of obesity among workers is of importance and has been widely studied [47], this review focused on the obesity effect on occupationally-relevant outcomes.

## Conclusion

Obesity is a serious global public health threat. In order to build up a comprehensive profile of its effects, it is crucial to have easy-to-use yet reliable measures that allow for classification of individuals into distinct risk groups. A large body of research has been conducted in the occupational health field regarding obesity. Use of indirect measures such as BMI may be justifiable in large scale public health studies due to their ease of use and low cost. However, due to limitations of these measures, cautious use of them is suggested as the sole obesity measure in small-scale observational studies.

## Abbreviations

%BF: Body Fat Percentage
BIA: Bioelectrical Impedance Analysis
BMI: Body Mass Index
DEXA: Energy X-ray Absorptiometry
GF: General Physical or Mental Work-Related Functioning
LBP: Low Back Pain
NIH: National Institute of Health
OA: Osteoarthritis
OW/OB: Overweight/Obese
RoM: Range of Motion
ST: Skinfold Thickness
TBS: Task or Body Part Specific Functioning
WC: Waist Circumference
WHO: World Health Organization
WHR: Waist–Hip Ratio
